# Development of Antimicrobial Stapled Peptides Based on Magainin 2 Sequence

**DOI:** 10.3390/molecules26020444

**Published:** 2021-01-16

**Authors:** Motoharu Hirano, Chihiro Saito, Hidetomo Yokoo, Chihiro Goto, Ryuji Kawano, Takashi Misawa, Yosuke Demizu

**Affiliations:** 1 Division of Organic Chemistry, National Institute of Health Sciences, 3-25-26, Tonomachi, Kawasaki, Kanagawa 210-9501, Japan; w205435g@yokohama-cu.ac.jp (M.H.); yokoo@nihs.go.jp (H.Y.); w195412a@yokohama-cu.ac.jp (C.G.); 2 Graduate School of medical Life Science, Yokohama City University, 1-7-29, Yokohama, Kanagawa 230-0045, Japan; 3 Department of Biotechnology and Life Science, Tokyo University of Agriculture and Technology, 2-24-6 Naka-cho, Koganei, Tokyo 184-8588, Japan; c-saito@st.go.tuat.ac.jp (C.S.); rjkawano@cc.tuat.ac.jp (R.K.)

**Keywords:** antimicrobial peptides, magainin 2, stapled peptide, helical structure, amphipathicity

## Abstract

Magainin 2 (**Mag2**), which was isolated from the skin of the African clawed frog, is a representative antimicrobial peptide (AMP) that exerts antimicrobial activity via microbial membrane disruption. It has been reported that the helicity and amphipathicity of **Mag2** play important roles in its antimicrobial activity. We investigated and recently reported that 17 amino acid residues of **Mag2** are required for its antimicrobial activity, and accordingly developed antimicrobial foldamers containing α,α-disubstituted amino acid residues. In this study, we further designed and synthesized a set of **Mag2** derivatives bearing the hydrocarbon stapling side chain for helix stabilization. The preferred secondary structures, antimicrobial activities, and cell-membrane disruption activities of the synthesized peptides were evaluated. Our analyses revealed that hydrocarbon stapling strongly stabilized the helical structure of the peptides and enhanced their antimicrobial activity. Moreover, peptide **2** stapling between the first and fifth position from the *N*-terminus showed higher antimicrobial activity than that of **Mag2** against both gram-positive and gram-negative bacteria without exerting significant hemolytic activity. To investigate the modes of action of tested peptides **2** and **8** in antimicrobial and hemolytic activity, electrophysiological measurements were performed.

## 1. Introduction

**Figure 1:**
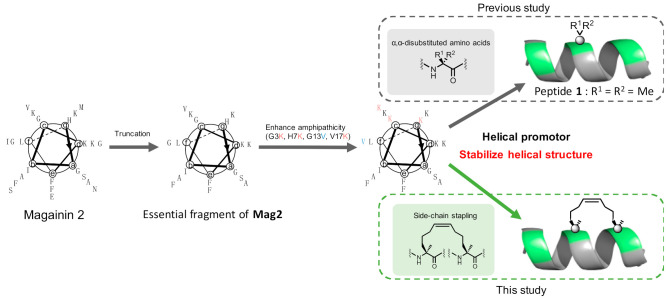
The peptide design based on the essential fragment of magainin 2 (**Mag2**).

Magainin 2 (**Mag2**), a representative antimicrobial peptide (AMP), plays an important role in the natural immune system and has recently received attention as a next-generation drug for multi-drug resistance bacteria [1,2]. **Mag2** comprises cationic and hydrophobic amino acid residues and employs a helical structure in aqueous solution. These properties are required for interaction with microbial membranes, and subsequent **Mag2** insertions into membranes induce bacterial pore formation and ion efflux to exert their antimicrobial activity. The antimicrobial activity of AMPs targets the bacterial membrane; thus, resistance against AMPs can hardly be generated by their long-term administration [3,4]. We have recently reported that 17 amino acid residues from the *N*-terminus of **Mag2** are essential for anti-microbial activity, and further structural development to stabilize its secondary structure was performed [5]. The introduction of α,α-disubstituted amino acids (dAAs), β-amino acids, and side-chain stapling into oligopeptides is useful for stabilizing the helical structure of oligopeptides, enhancing their biological functions, and exerting resistance against digestive enzymes like proteases [6,7]. To achieve this, dAAs were inserted into the active fragment of **Mag2**. Peptide **1** contains two Aib (X) residues, a representative dAA that works as a helical stabilizer and exhibits potent antimicrobial activity against both gram-positive and gram-negative microbes without any significant hemolytic activity against human red blood cells (RBCs) [5]. Furthermore, peptide **1** showed resistance against digestive enzymes due to the unnatural amino acid residues. These data suggest that further structural developments are required to stabilize the helical structure of the essential fragment of **Mag2**; this may be achieved by using non-proteinogenic amino acids as a promising strategy for increasing antimicrobial activity. The introduction of a hydrocarbon stapling side chain into a peptide sequence could also improve its bioactivity and stability against several proteases [8,9,10]. Walensky has recently reported that **Mag2** derivatives that contain the stapling side chain demonstrated potent antimicrobial activity against broad microbes without cytotoxicity in vivo [11]. The report indicated that introducing the stapling side chain into the essential fragment of **Mag2** is a promising strategy for improving its antimicrobial activity. Conversely, it has been reported that the *N*-terminal fatty acylation of AMPs enhances its affinity to microbial membranes, which increases its antimicrobial activity [12]. Moreover, fatty acylation promotes the micelle formation of peptides and also reduces their hemolytic activity [13]. In this report, we performed a structural development of peptide **1** to enhance its antimicrobial activity by introducing side-chain stapling (Figure 1). Thus, we designed several peptides bearing the stapling side chain and synthesized them in the hydrophobic regions and the *N*-terminal fatty acyl group and subsequently evaluated their preferred secondary structures, antimicrobial activity against gram-positive and gram-negative bacteria, and hemolytic activity against human RBCs.

## 2. Results

First, we designed and synthesized peptides **2**–**9** bearing the stapling side chain. Generally, suitable (S)-pentenyl alanine and/or (R)-octenyl alanine are introduced at the *i*/*i* + 4 or *i*/*i* + 7 position. The effects of the position and length of the stapling side chain on their preferred secondary structures and antimicrobial activity were investigated. The designated peptides were synthesized using microwave-assisted Fmoc-based solid-phase peptide synthesis method and olefin metathesis using second-generation Grubbs catalysis under an N_2_ atmosphere. The peptides were cleaved from the resin and precipitated using cold ether, and the resulting residues were purified using reverse-phase high-performance liquid chromatography (HPLC); from this, the target peptide was identified with liquid phase chromatography–mass spectrometry (LC-MS).

Next, we investigated the antimicrobial activity of the synthesized peptides **2**–**9** against gram-positive *Staphylococcus aureus* and gram-negative *Escherichia coli* DH5α, *Pseudomonas aeruginosa*, and multiple-drug resistant *P. aeruginosa* (MDRP). The minimum inhibitory concentration (MIC) against both gram-positive and gram-negative bacteria was measured as the antimicrobial activity. As shown in Table 1, peptides **2**–**6** with *i*/*i* + 4 position stapling showed higher antimicrobial activity against both gram-positive and gram-negative bacteria compared to **Mag2** and peptide **1**. Meanwhile, peptides **7**–**9** with *i*/*i* + 7 position stapling exhibited lower or the same extent of antimicrobial activity compared to peptides **2**–**6**. These results indicated that the introduction of a stapled side chain at the *i*/*i* + 7 position tends to decrease the antimicrobial activity. Hemolytic activity against human red blood cells (RBCs) was also investigated to assess the cytotoxicity. Peptide **2** showed potent antimicrobial activity without hemolytic activity, whereas peptides **3**–**6** exhibited significant hemolytic activity. Moreover, peptides **7**–**9** with *i*/*i* + 7 positional stapling showed strong hemolytic activity, among which peptide **8** exhibited extremely high hemolytic activity.

As mentioned above, it has been reported that N-terminus fatty acylation could increase antimicrobial activity and decrease hemolytic activity. Based on this, we designed and synthesized N-terminal acylated derivatives based on peptide **2** and evaluated the effects of the length of acyl groups on antimicrobial and hemolytic activity. As shown in Table 2, peptide **C6-2**, which has a hexyl group, showed the same level of antimicrobial activity against both gram-positive and gram-negative bacteria as did peptide **2**, whereas the introduction of the N-terminal hexyl group increased its hemolytic activity. Moreover, the introduction of a lauryl or stearyl group decreased antimicrobial activity and increased hemolytic activity. These data demonstrated that the increase of hydrophobicity promoted not only antimicrobial activity but also hemolytic activity.

**Figure 2:**
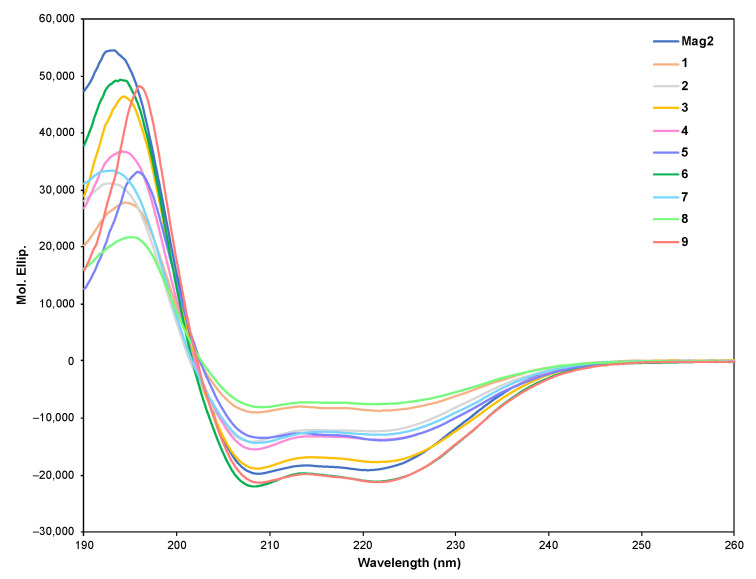
Preferred secondary structural analysis of **Mag2** and peptides **1–9** in pH = 7.4 phosphate buffered saline solution with 1% sodium dodecyl sulphate. The mean residue ellipticity was plotted against wavelength. The values from three scans were averaged per sample. Peptide concentration = 100 μM.

Next, we performed preferred secondary structural analyses of peptides **1**–**9** in pH = 7.4 phosphate buffered saline (PBS) containing a 1% sodium dodecyl sulphate (SDS) solution, which has been reported to mimic the cell membrane environment [14,15]. As shown in Figure 2, all peptides with side-chain stapling showed negative maxima at around 208 and 222 nm, indicating that the peptides formed a stabilized helical structure. These results revealed that the introduction of a stapled side chain into the peptide sequence stabilized its helical structure, and the positioning of the stapled side chain did not affect their preferred secondary structure. Furthermore, we evaluated the effects of the long fatty acyl groups on their secondary structures. As shown in Figure S1, peptides **C6-2** and **C12-2** showed a curve similar to that of peptide **2.** The circular dichroism (CD) spectral analysis of **C18-2** could not be performed because of its poor solubility in the aqueous buffer solution.

**Figure 3:**
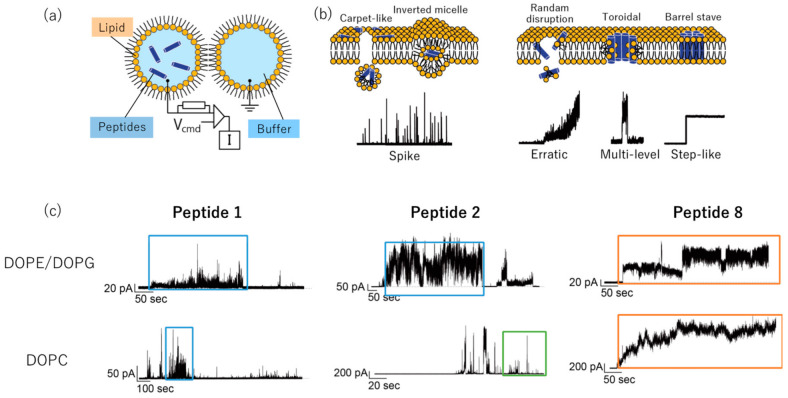
(**a**) Electrophysiological measurement [18]. (**b**) Typical current patterns with the treatment of cell penetrating peptides (CPPs) and antimicrobial peptides (AMPs) [18]. (**c**) The electrophysiological signals during treatment of peptides **1**, **2,** and **8** at 100 nM.

The electrophysiological measurement of AMPs could be a useful method for observing and analyzing pore formation using the droplet contact method (Figure 3a,b) [16,17]. The representative observations of electrophysiological measurements are shown in Figure 3c. Briefly, cell penetrating peptides (CPPs) exhibit step-like signals [16]. In contrast, the toroidal pore model exhibits multilevel current signals [17,18]. Based on this knowledge, we performed electrophysiological measurement against 1,2-dioleoyl-*sn*-glycero-3-phosphoethanolamine (DOPE)/1,2-dioleoyl-*sn*-glycero-3-phospho-(1′-*rac*-glycerol) (DOPG) and 1,2-dioleoyl-*sn*-glycero-3-phosphocholine (DOPC) bilayer membranes, which mimic the microbial and mammalian cell membranes, respectively, to investigate the action mechanisms on the antimicrobial and hemolytic activities of peptides **1**, **2**, and **8**. As shown in Figure 3c, peptide **1** exhibited membrane disruption activity against the DOPE/DOPG bilayer membrane, whereas a successive increase of current signals which should indicate the pore formation was observed by the treatment of peptides **2** and **8** at 100 nM. Conversely, electrophysiological measurement using the DOPC bilayer membrane revealed that peptide **2** showed spike signals, which indicates its cell membrane permeability against the DOPC membrane. 

**Figure 4:**
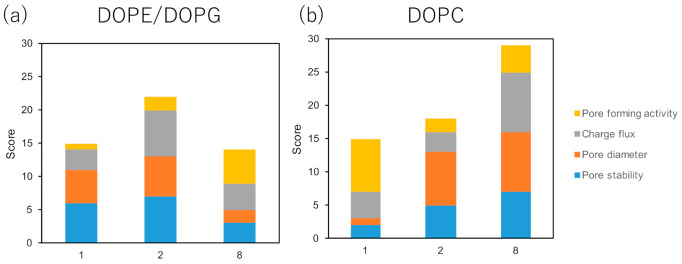
Scores for peptides **1**, **2**, and **8** used for the pore-formation activity analysis. The total score of pore formation, charge flux, pore diameter, and pore stability against (**a**) 1,2-dioleoyl-*sn*-glycero-3-phosphoethanolamine (DOPE)/1,2-dioleoyl-*sn*-glycero-3-phospho-(1′-*rac*-glycerol) (DOPG) and (**b**) 1,2-dioleoyl-*sn*-glycero-3-phosphocholine (DOPC) membrane was evaluated for peptides **1, 2,** and **8**.

Furthermore, treatment of peptide **8** showed a subsequent increase in current signals, which indicates that this peptide could form a pore in the DOPC membrane. It has been reported that the antimicrobial activities of AMPs are positively correlated with the scores for each pore-forming parameter, including pore stability, pore diameter, charge flux, and pore-forming activity [18]. Each parameter was evaluated as previously reported [18]. The total score of peptides **1**, **2**, and **8** was determined to detect their pore-forming activity. As shown in Figure 4, the score of peptide **2** against the DOPE/DOPG bilayer membrane was higher than those of peptides **1** and **8**, which indicates that peptide **2** exerted antimicrobial activity against the microbial membrane via pore formation. Further, peptide **8** exhibited a higher score against the DOPC bilayer membrane, whereas the score of peptide **1** was the lowest among the tested peptides. These results demonstrate that peptide **2** formed pores on the microbial membrane but not so on the mammalian membrane.

## 3. Conclusions

In this study, we designed and synthesized a series of antimicrobial peptides based on **Mag2** and its essential fragment by introducing a hydrocarbon stapling side chain. Synthesized peptides **2**–**9** were evaluated for their antimicrobial activities against gram-positive and gram-negative bacteria and their preferred secondary structures were investigated by measuring the CD spectra. Peptides **2**–**6** with a stapling side chain at the *i*/*i + 4* positions showed potent antimicrobial activities against both gram-positive and gram-negative bacteria. Among these, peptide **2** with *N*-terminal stapling showed lower hemolytic activity compared with other peptides, particularly, peptides **7**–**9** and *N*-acylated peptides **C6-2**, **C12-2**, and **C18-2**, thus indicating that an increase in hydrophobicity enhances hemolytic activity. CD spectral analysis revealed that peptides **2**–**9** with *i*/*i + 4* or *i*/*i + 7* positioning adopted stable helical structures in aqueous solution. Furthermore, an electrophysiological analysis was conducted to assess the pore-forming activity of peptides **1**, **2**, and **8**. The results revealed that peptide **2** showed a prominent score against the DOPE/DOPG membrane, which mimics the microbial membrane, but showed a lower score against the DOPC membrane, which mimics the mammalian membrane. Alternatively, peptide **8**, which showed the highest hemolytic activity, exhibited a significantly higher score against the DOPC membrane. The scoring method could be a useful tool for predicting AMPs properties.

In conclusion, we developed novel AMPs based on **Mag2** essential fragments by introducing hydrocarbon side-chain stapling. Peptide **2** showed potent antimicrobial activity against both gram-positive and gram-negative bacteria without significant hemolytic activity. These results suggest that peptide **2** could be a promising reagent for the treatment of MDRPs. Moreover, the electrophysiological measurements revealed that antimicrobial activity and hemolytic activity were positively correlated with the total score of pore-forming properties against the DOPE/DOPG and DOPC bilayer membrane. Based on our scoring methods, further structural development of peptide **2** could lead to novel AMPs with broad antimicrobial spectra, including MDRPs. 

## 4. Materials and Methods

### 4.1. General Information

Chemicals were purchased from Sigma-Aldrich (St. Louis, MO, USA); Kanto Chemicals (Tokyo, Japan); Tokyo Chemical Industry (Tokyo, Japan); Wako Pure Chemical Industries, (Osaka, Japan); and Watanabe Chemical Industries, (Hiroshima, Japan); and were used without further purification. Mass spectra were obtained using Shimadzu IT-TOF MS (Shimadzu, Kyoto, Japan) equipped with an electrospray ionization source.

### 4.2. Peptide Synthesis

The designed peptides were synthesized by Fmoc-based solid-phase methods using Liberty Blue (CEM Corp. Matthews, NC, USA). A representative coupling and deprotection cycle is described as follows: ProTide™ resin was soaked for 30 min in CH_2_Cl_2_. After washing with dimethylformamide (DMF), Fmoc-amino acid (4 equiv.), Oxyma (4 equiv.), and dissolved inorganic carbon (DIC; 10 equiv.) in a solution of DMF were added to the resin. Fmoc protective groups were deprotected using 20% piperidine in DMF. Ring-closing metathesis reactions were performed using second-generation Grubb’s catalyst under N_2_ gas bubbling condition. The resin was suspended in a cleavage cocktail (95% trifluoroacetic acid [TFA], water 2.5%, 2.5% triisopropylsilane) at room temperature for 3 h. TFA was evaporated to a small volume under a stream of N_2_ and dripped into cold ether to precipitate the peptide. The peptides were dissolved in dimethyl sulfoxide and purified using reverse-phase HPLC using a Discovery^®^ BIO Wide Pore C18 column (Supelco, Bellefonte, PA, USA) (25 cm × 21.2 mm solvent A: 0.1% TFA/water, solvent B: 0.1% TFA/MeCN, flow rate: 10.0 mL × mL^−1^, gradient: 10–90% gradient of solvent B over 30 min). After purification, the peptide solutions were lyophilized, and peptide purity was assessed using analytical HPLC and a Discovery^®^ BIO Wide Pore C18 column (25 cm × 4.6 mm; solvent A: 0.1% TFA/water, solvent B: 0.1% TFA/MeCN, flow rate: 1.0 mL × mL^–1^, gradient: 10–100% gradient of solvent B over 30 min).

### 4.3. CD Spectrometry

CD spectra were recorded using J-1100 CD spectrometer (Jasco, Tokyo, Japan) with a 1.0-mm path length cell under 25 °C. The data are expressed in terms of [θ]; that is, total molar ellipticity (deg cm^2^ dmol^−1^). Peptides were dissolved in 20 mM PBS solution (pH = 7.4) with 1% SDS at concentrations of 100 µM. 

### 4.4. Antimicrobial Activity

Selected bacterial strains were obtained from Biological Resource Center, NITE (NBRC; Tokyo, Japan). *E. coli* DH5α was purchased from BioDynamics Laboratory, Inc. (Tokyo, Japan). The antimicrobial activities of the peptides against the gram-positive *S. aureus* and gram-negative *E. coli* DH5α, *P. aeruginosa*, and MDRP were measured using standard broth microdilution method as previously described [19]. Briefly, the bacteria were inoculated and grown overnight at 37 °C on agar media for other organisms i and then collected with liquid media for other organisms ii, according to Japanese Pharmacopoeia, 17th Edition. Each peptide was 2-fold serially diluted with an initial concentration of 500 μM to a final concentration of 1 μM using PBS. Then, 10 μL of the peptide solution was added to each well of a sterile 96-well plate. Subsequently, 90 μL of inoculation with 10^4^ colony forming units (CFUs) per mL were added to each well, and the plate was incubated at 35 °C for 16 h. MIC was defined as the lowest concentration of the peptide at which bacterial growth was completely inhibited based on visual observation at 595 nm.

### 4.5. Hemolysis Activity

Human RBCs were kindly supplied by the Japanese Red Cross Society (Tokyo, Japan) after collection from volunteers under informed consent. The hemolysis test was performed using a previously reported method [20]. In brief, the RBCs were washed three times and resuspended in 172 mM Tris-HCl buffer (pH = 7.6), then, 50 µL of the RBC solution was incubated with 50 µL of each peptide at 37 °C for 30 min. The suspensions were centrifuged at 400 rpm for 5 min, and the absorbance of the supernatant was measured at 535 nm. M-Lycotoxin [21] served as the positive control.

### 4.6. Channel Current Measurement

Planar lipid bilayers were prepared as previously reported [22]. We used a micropipette (Eppendorf, Hamburg, Germany) at fabrication for lipid bilayers in each chamber. After addition of 2.3 μL of 10 mg/mL DOPE/DOPG (3:1 mol/mol) in *n*-decane solution to each chamber, we added 4.7 μL of 10 μM peptide liquid and 200 mM KCl liquid solution, allowing us to easily prepare the planar lipid bilayer.

Channel current signals were acquired using a JET patch-clam amplifier (Tecella, CA, USA). The droplet contact device was fabricated in our laboratory. It has two electrodes; one side was connected to the JET patch-clamp amplifier and applied 100 mV of constant voltage, and another side was grounded. As AMPs formed pores on the lipid bilayer, ions passed through the pores, resulting to show the current signals. We measured current signals under the conditions of 20 kHz of the sampling rate with 4 kHz of a low-pass filter at room temperature (23 ± 1 °C). Data were analyzed using pCLAMP ver. 10.5 (Molecular Devices, CA, USA) and Excel (Microsoft, Washington, USA). The classification of current signals obtained from several minutes to around 1 h data was performed according to the previous definition [16,17,18] using the same data numbers (N > 3, n > 150). Each parameter of the score for antimicrobial activities is summarized in Tables S1 and S2.

## Figures and Tables

**Table 1:** Peptide sequences of **Mag2** and tested peptides **1**–**9**, antimicrobial activity against gram-positive and gram-negative bacteria, and hemolytic activity against red blood cells (RBCs). X indicates the replacement of amino acid residue with Aib residues.

Peptide	Sequence	MIC (µM)				Hemolysis (µM)
		Gram Positive	Gram Negative			
		*S.a.*	*E.c.* (DH5α)	*P.a.*	MDRP	
**Mag2**	H-GIGKFLHSAKKFGKAFVGEIMNS-NH_2_	100	3.125	12.5	12.5	>100
**1**	H-GIKKFLKSXKKFVKXFK-NH_2_	12.5	3.125	3.125	3.125	50
**2**	H-**S_5_**IKK**S_5_**LKSAKKFVKAFK-NH_2_	3.125	1.56	1.56	1.56	50
**3**	H-GIKK**S_5_**LKS**S_5_**KKFVKAFK-NH_2_	3.125	1.56	3.125	0.78	6.25
**4**	H-GIKKFLK**S_5_**AKK**S_5_**VKAFK-NH_2_	6.25	1.56	1.56	0.78	3.125
**5**	H-GIKKFLKS**S_5_**KKF**S_5_**KAFK-NH_2_	12.5	1.56	6.25	3.125	1.56
**6**	H-GIKKFLKSAKK**S_5_**VKA**S_5_**K-NH_2_	3.125	1.56	1.56	0.78	3.125
**7**	H-GIKK**R_8_**LKSAKK**S_5_**VKAFK-NH_2_	25	3.125	3.125	1.56	6.25
**8**	H-GIKKFLK**R_8_**AKKFVK**S_5_**FK-NH_2_	>50	6.25	>50	3.125	0.19
**9**	H-GIKKFLKS**R_8_**KKFVKA**S_5_**K-NH_2_	6.25	3.125	6.25	3.125	3.125

**S_5_**_:_ (S)-4-pentenyl alanine; **R_8_**_,_ (R)-7-octenyl alanine; MIC, minimal inhibitory concentration; S.a., *Staphylococcus aureus*; E.C., *Escherichia coli* DH5α; P.a., *Pseudomonas aeruginosa*; MDRP, multiple-drug resistant *P. aeruginosa*.

**Table 2:** Antimicrobial activity and hemolytic activity of N-terminal fatty acylated peptides.

Peptide	Sequence	MIC (µM)				Hemolysis (µM)
		Gram Positive	Gram Negative			
		*S.a.*	*E.c.* (DH5α)	*P.a.*	MDRP	
**2**	H-**S_5_**IKK**S_5_**LKSAKKFVKAFK-NH_2_	3.125	1.56	1.56	1.56	50
**C6-2**	CH_3_(CH_2_)_4_CO-**S_5_**IKK**S_5_**LKSAKKFVKAFK-NH_2_	1.56	1.56	3.125	3.125	12.5
**C12-2**	CH_3_(CH_2_)_10_CO-**S_5_**IKK**S_5_**LKSAKKFVKAFK-NH_2_	6.25	6.25	12.5	25	0.78
**C18-2**	CH_3_(CH_2_)_16_CO-**S_5_**IKK**S_5_**LKSAKKFVKAFK-NH_2_	>50	>50	>50	>50	0.78

## Data Availability

The data presented in this study are available on request from the corresponding author.

## Supplementary Materials

The following are available online. Figure S1: CD spectra analysis of *N*-acylated peptides; Table S1: The pore forming score for antimicrobial acitivities of peptides **1**, **2**, and **8** in DOPE/DOPG membrane; Table S2: The pore forming score for antimicrobial acitivities of peptides **1**, **2**, and **8** in DOPC membrane.

## Sample Availability

Samples of the compounds **1**–**9** are available from the authors.
