# Supplementary material for: Development of Antimicrobial Stapled Peptides Based on Magainin 2 Sequence
Source: Molecules. 2021 Jan 16;26(2):444. doi: 10.3390/molecules26020444 (PMC7830303; doi:10.3390/molecules26020444)
Supplement: Supplementary file 1 [file molecules-26-00444-s001.pdf]

Development of Antimicrobial Stapled Peptides Based on Magainin 2 Sequence

Motoharu Hirano, [1,2] **Chihiro Saito,** [3] **Hidetomo Yokoo,** [1] **Chihiro Goto,** [1,2] **Ryuji Kawano,** [3] **Takashi Misawa,** [1,*] **Yosuke Demizu** [1,2,*]

[1] National Institute of Health Sciences, 3-25-26, Tonomachi, Kawasaki, Kanagawa 210-9501, Japan
[2] Graduate School of medical Life Science, Yokohama City University, 1-7-29, Yokohama, Kanagawa 230-0045, Japan
[3] Department of Biotechnology and Life Science, Tokyo University of Agriculture and Technology, 2-24-6 Naka-cho, Koganei, Tokyo 184-8588, Japan

* Correspondence: demizu@nihs.go.jp (Y.D.); misawa@nihs.go.jp (T.M.); Tel.:+81-44-270-6578

Table of Contents

Figure S1 CD spectra analysis of *N*-acylated peptides S3

ESI-TOF-MS and HPLC data of the synthesized peptides S4

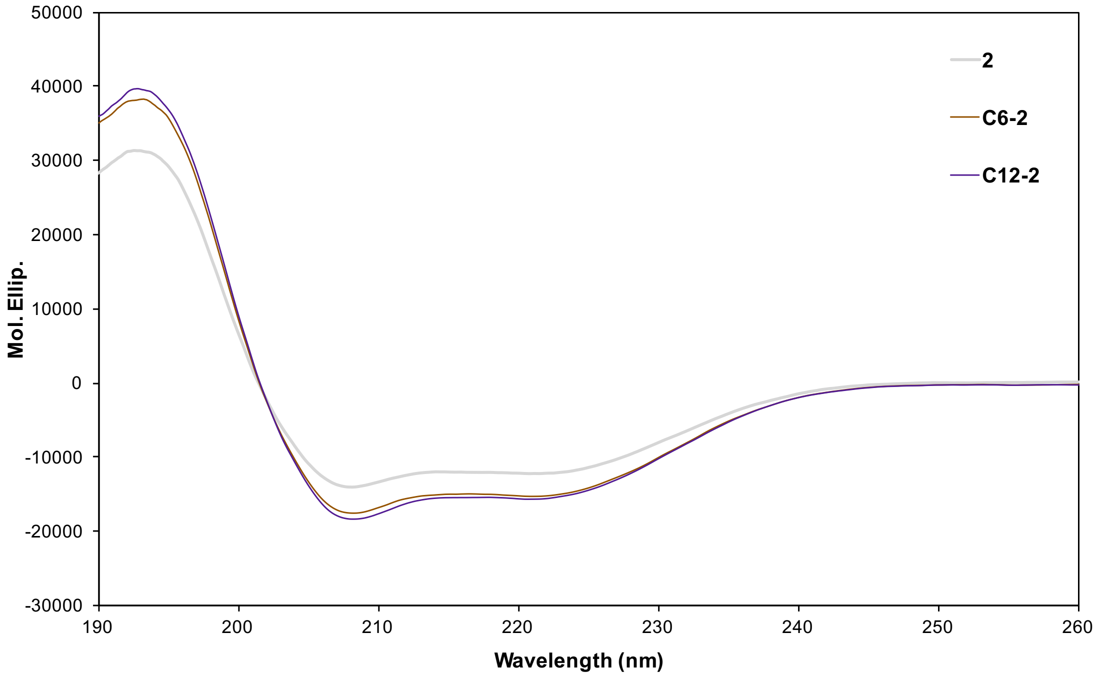

Figure S1. Preferred secondary structure of peptide **2, C6-2, and C12-2** in pH = 7.4 PBS solution with 1% SDS. Peptide concentration = 100 μM.

Mag2

H-Gly-Ile-Gly-Lys-Phe-Leu-His-Ser-Ala-Lys-Lys-Phe-Gly-Lys-Ala-Phe-Val-Gly-Glu-Ile-Met-Asn-Ser -NH_2

HPLC: Conditions = solvent A 0.1% TFA/water, solvent B 0.1% TFA/CH_3CN

Gradient = 10-90% gradient of solvent B over 30 min

Purity: 99.7%

HRMS (ESI^+) calcd for $C_{114}H_{181}N_{31}O_{28}S$ $[M+4H]^{4+}$: 617.0933; found: 617.0840

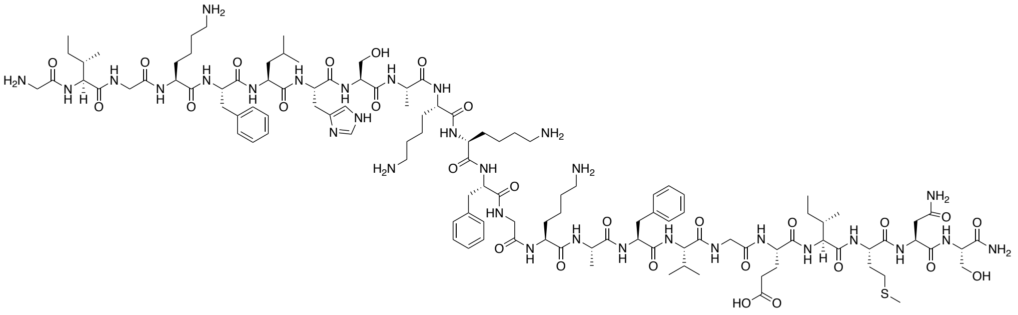

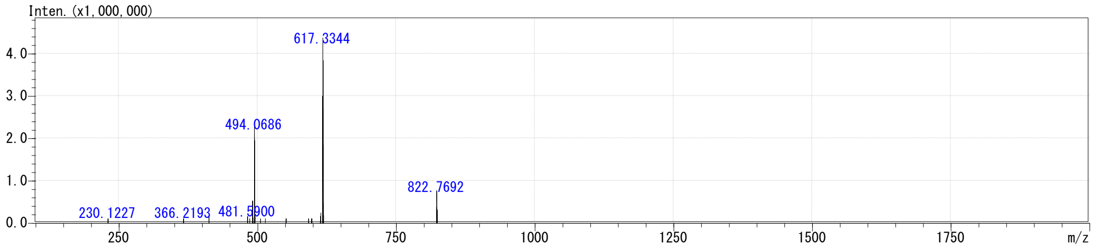

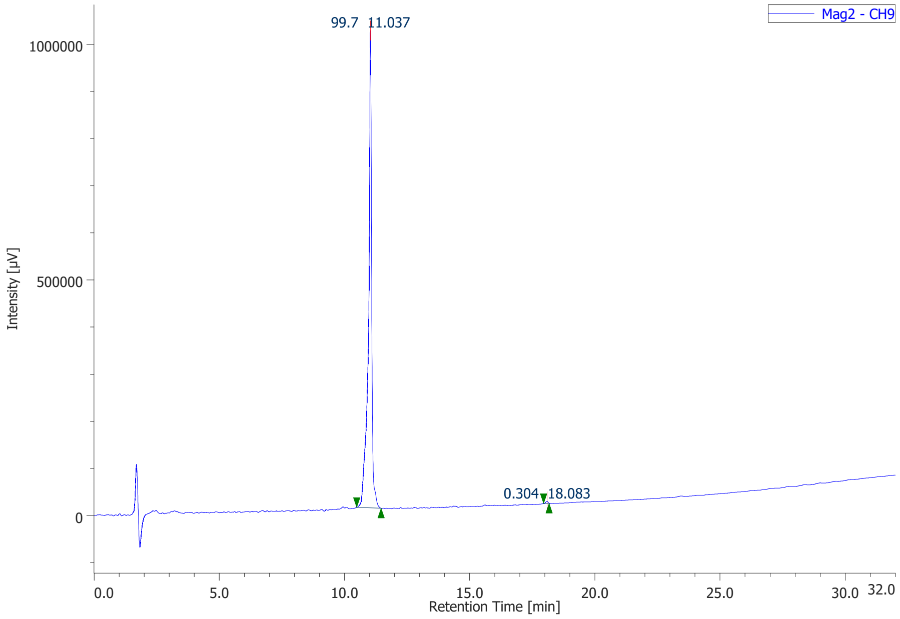

Peptide **1**

H-Gly-Ile-Lys-Lys-Phe-Leu-Lys-Ser-Aib-Lys-Lys-Phe-Val-Lys-Aib-Phe-Lys-NH_2

HPLC: Conditions = solvent A 0.1% TFA/water, solvent B 0.1% TFA/CH_3CN

Gradient = 10-90% gradient of solvent B over 30 min

Purity: 96.4%

HRMS (ESI^+) calcd for $C_{99}H_{167}N_{25}O_{18}$ $[M+5H]^{5+}$: 399.8662; found: 399.8595

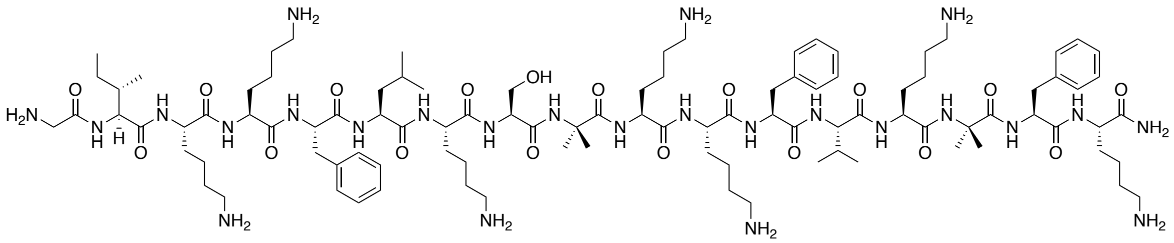

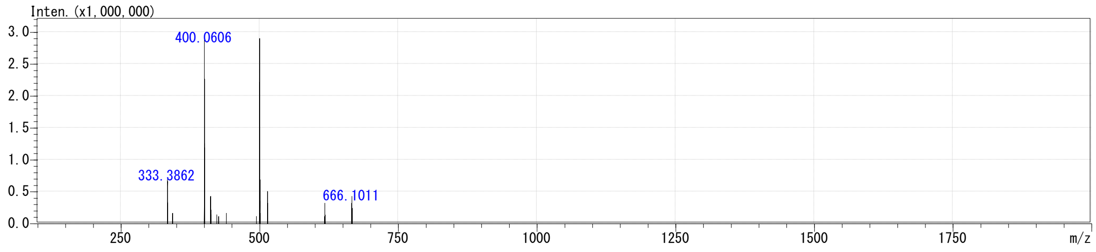

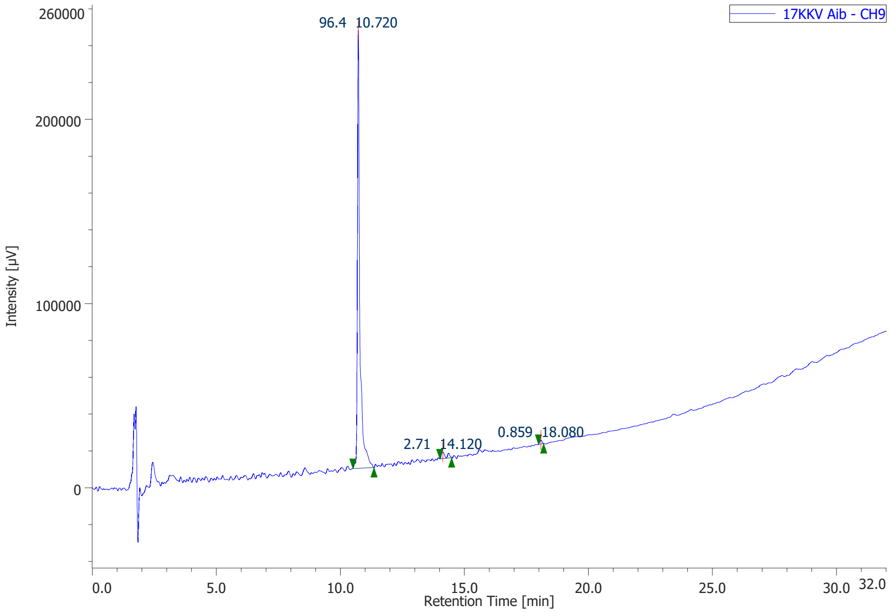

Peptide **2**

H-S_5-Ile-Lys-Lys-S_5-Leu-Lys-Ser-Ala-Lys-Lys-Phe-Val-Lys-Ala-Phe-Lys-NH_2

HPLC: Conditions = solvent A 0.1% TFA/water, solvent B 0.1% TFA/CH_3CN

Gradient = 10-90% gradient of solvent B over 30 min

Purity: 95.0%

HRMS (ESI$^+$) calcd for $C_{100}H_{173}N_{25}O_{18}$ $[M+5H]^{5+}$: 403.4756; found: 403.4797

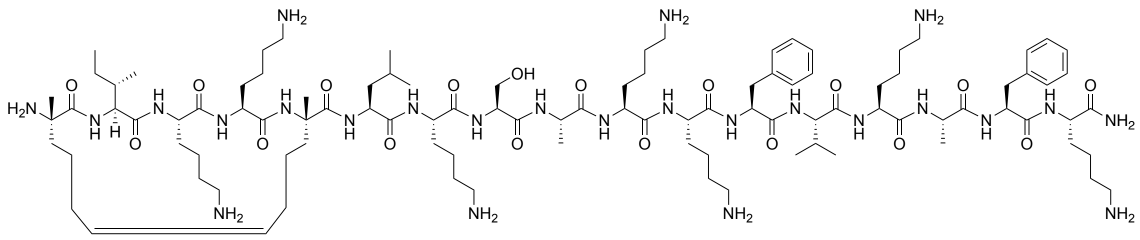

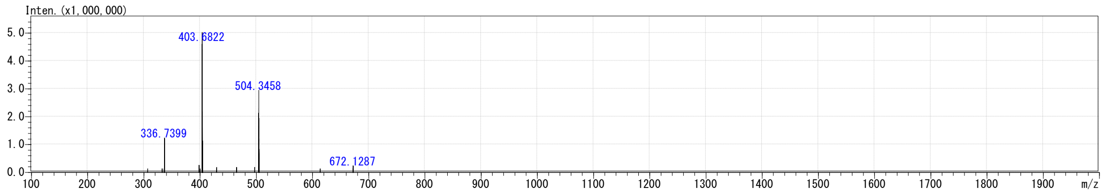

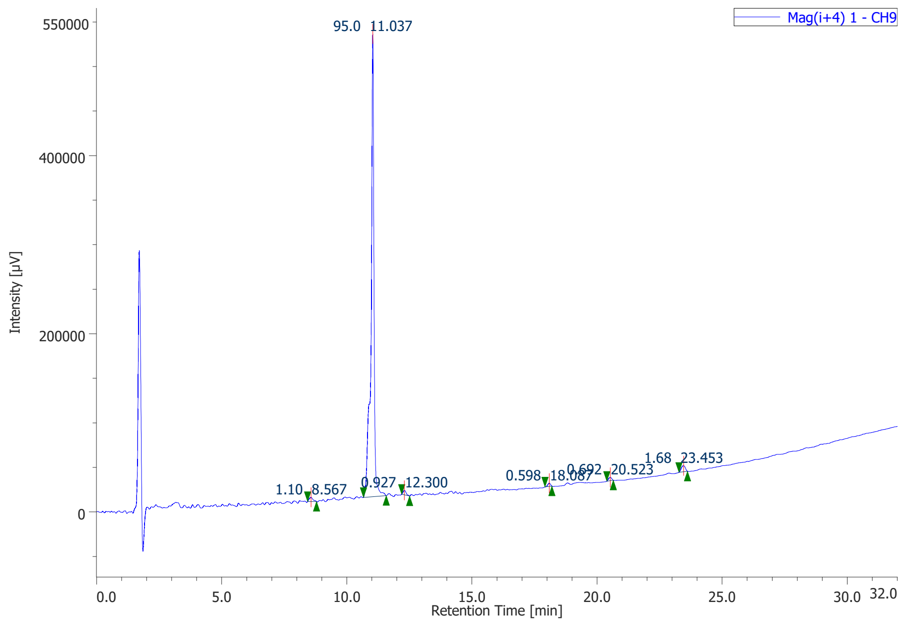

Peptide **3**

H-Gly-Ile-Lys-Lys-S_5-Leu-Lys-Ser-S_5-Lys-Lys-Phe-Val-Lys-Ala-Phe-Lys-NH_2

HPLC: Conditions = solvent A 0.1% TFA/water, solvent B 0.1% TFA/CH_3CN

Gradient = 10-90% gradient of solvent B over 30 min

Purity: 96.2%

HRMS (ESI$^+$) calcd for $C_{99}H_{171}N_{25}O_{18}$ $[M+5H]^{5+}$: 400.6725; found: 400.6795

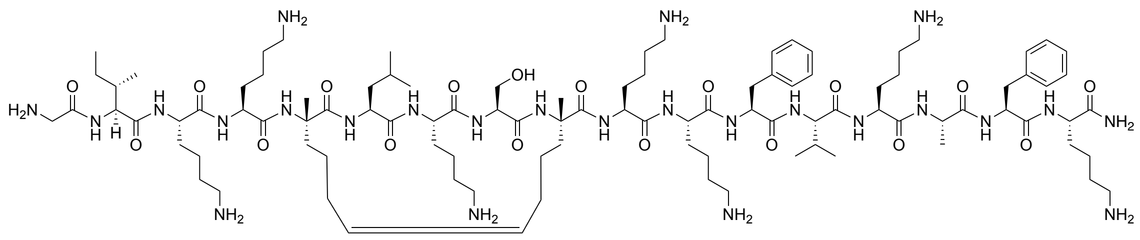

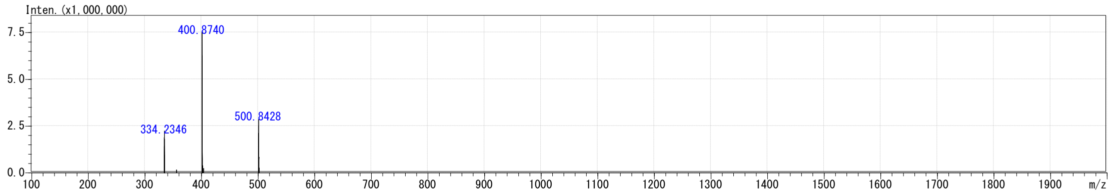

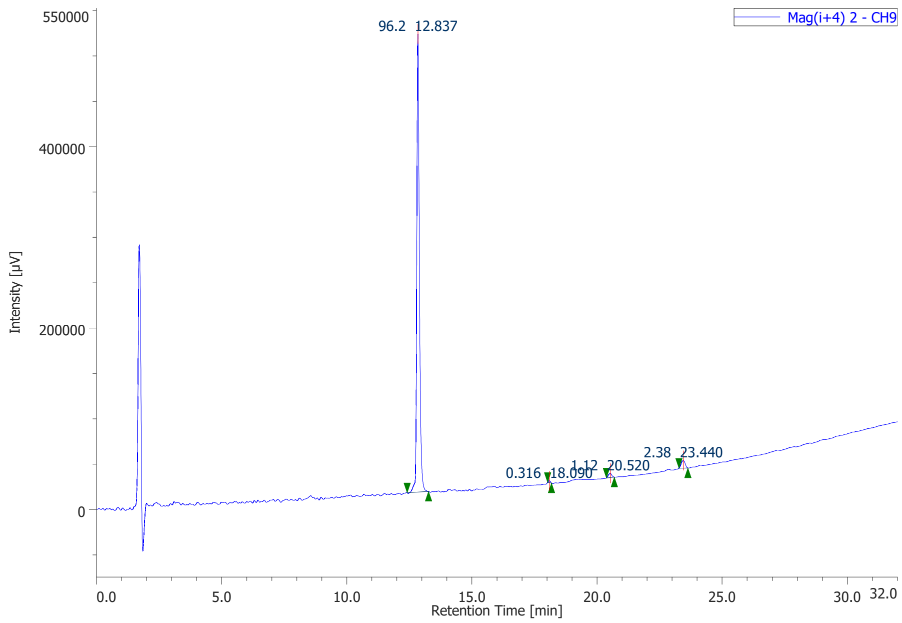

Peptide **4** (E/Z isomers were detected)

H-Gly-Ile-Lys-Lys-Phe-Leu-Lys-S_5-Ala-Lys-Lys-S_5-Val-Lys-Ala-Phe-Lys-NH_2

HPLC: Conditions = solvent A 0.1% TFA/water, solvent B 0.1% TFA/CH_3CN

Gradient = 10-90% gradient of solvent B over 30 min

Purity: 99.3%

HRMS (ESI$^+$) calcd for $C_{99}H_{171}N_{25}O_{17}$ $[M+5H]^{5+}$: 397.4735; found: 397.4748

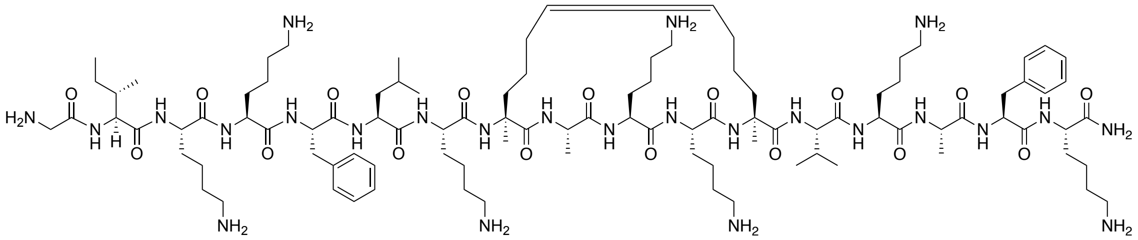

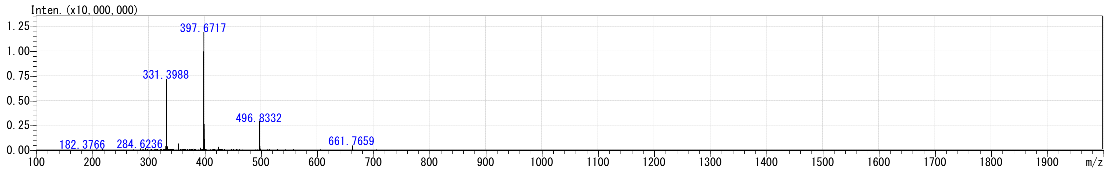

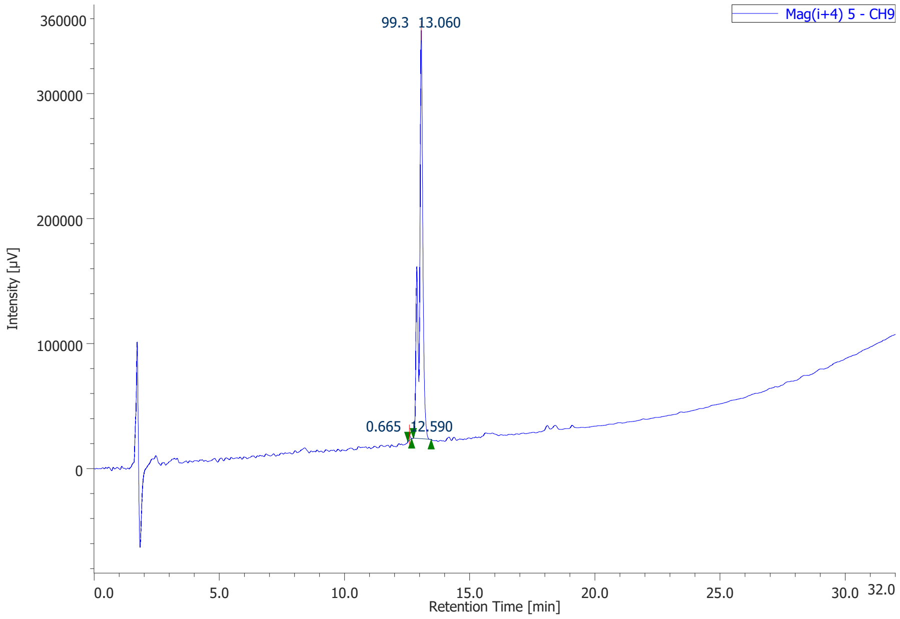

Peptide **5**

H-Gly-Ile-Lys-Lys-Phe-Leu-Lys-Ser-S_5-Lys-Lys-Phe-S_5-Lys-Ala-Phe-Lys-NH_2

HPLC: Conditions = solvent A 0.1% TFA/water, solvent B 0.1% TFA/CH_3CN

Gradient = 10-90% gradient of solvent B over 30 min

Purity: 99.8%

HRMS (ESI^+) calcd for $C_{103}H_{171}N_{25}O_{18}$ $[M+4H]^{4+}$: 512.5887; found: 512.5864

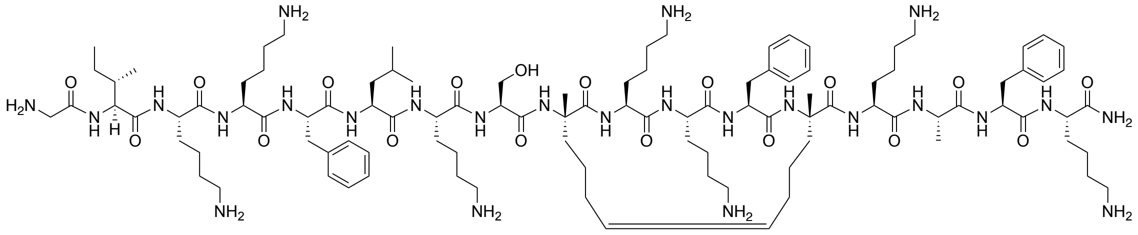

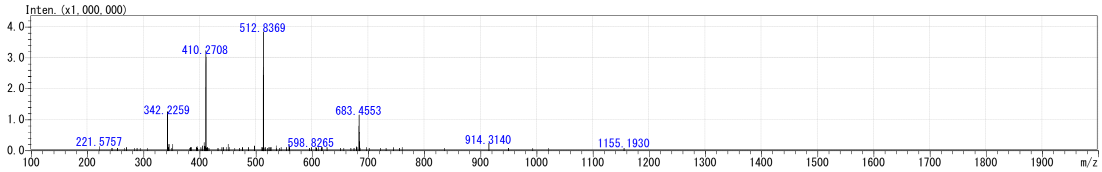

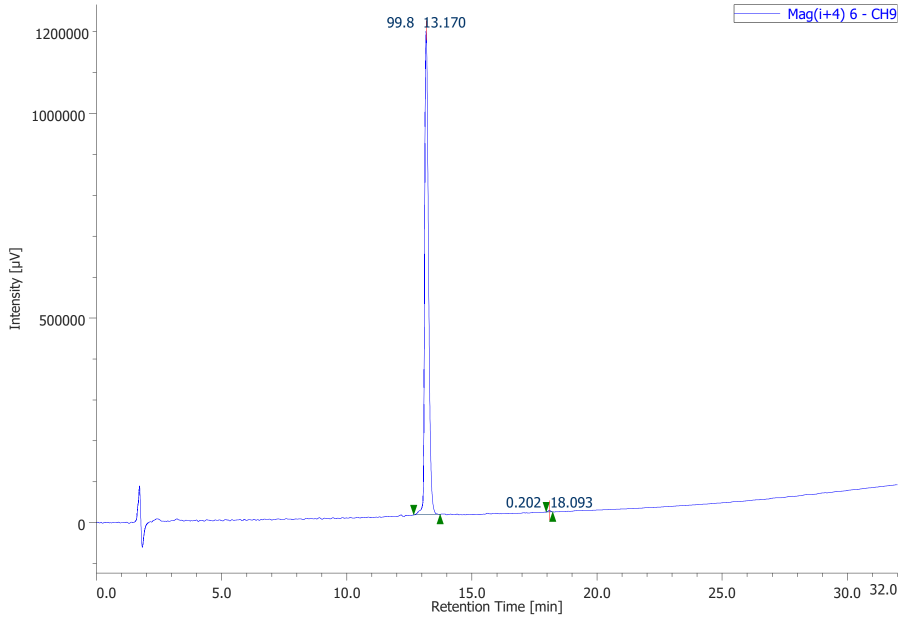

Peptide **6**

H-Gly-Ile-Lys-Lys-Phe-Leu-Lys-Ser-Ala-Lys-Lys-S_5-Val-Lys-Ala-S_5-Lys-NH_2

HPLC: Conditions = solvent A 0.1% TFA/water, solvent B 0.1% TFA/CH_3CN

Gradient = 10-90% gradient of solvent B over 30 min

Purity: 98.5%

HRMS (ESI$^+$) calcd for $C_{93}H_{167}N_{25}O_{18}$ $[M+5H]^{5+}$: 385.4662; found: 385.4703

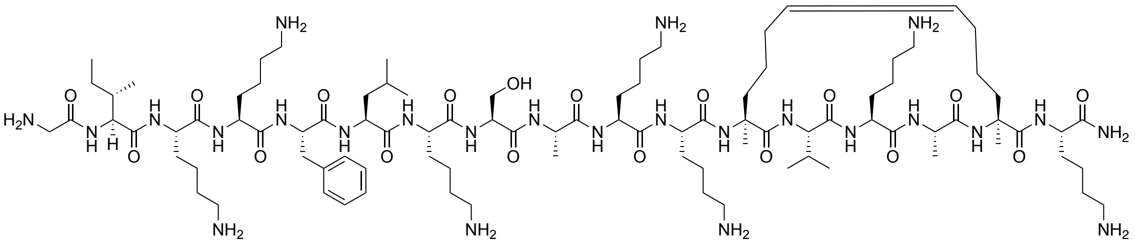

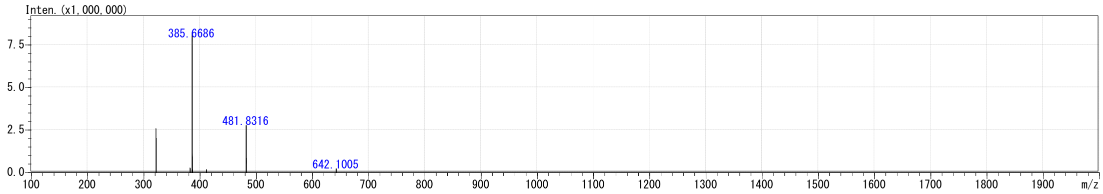

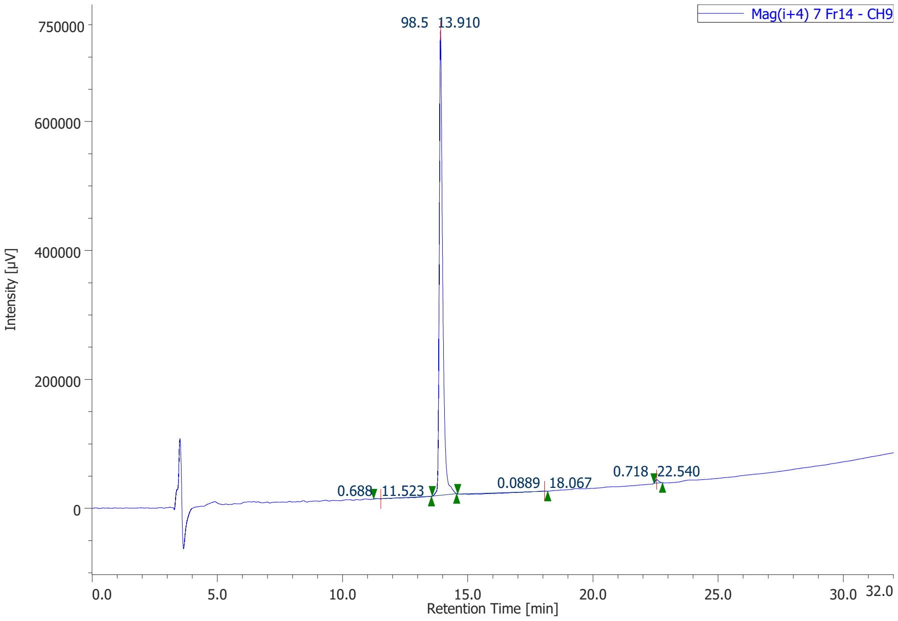

Peptide **7** (E/Z isomers were detected)

H-Gly-Ile-Lys-Lys-R_8-Leu-Lys-Ser-Ala-Lys-Lys-S_5-Val-Lys-Ala-Phe-Lys-NH_2

HPLC: Conditions = solvent A 0.1% TFA/water, solvent B 0.1% TFA/CH_3CN

Gradient = 10-90% gradient of solvent B over 30 min

Purity: 98.1%

HRMS (ESI^+) calcd for $C_{96}H_{173}N_{25}O_{18}$ $[M+3H]^{3+}$: 655.7875; found: 655.8010

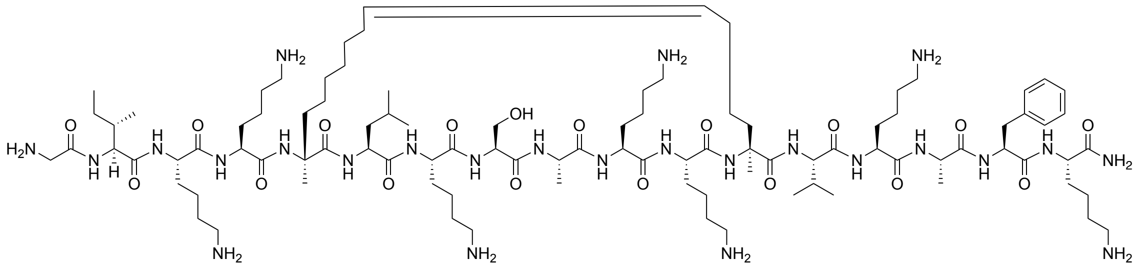

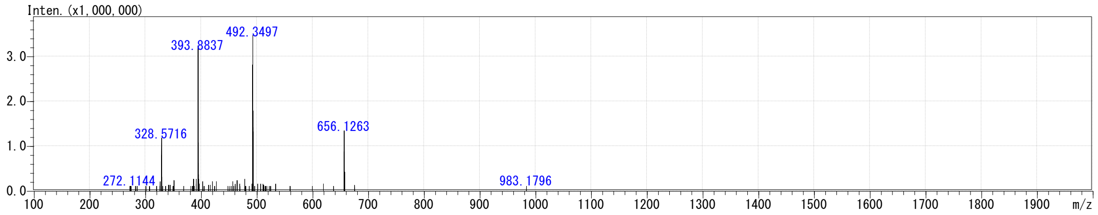

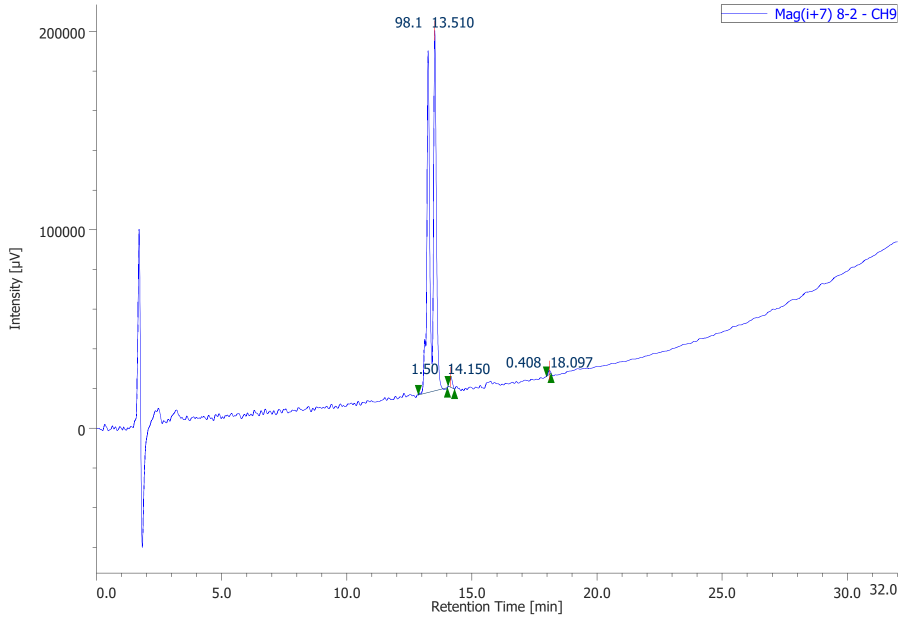

Peptide **8**

H-Gly-Ile-Lys-Lys-Phe-Leu-Lys-R_8-Ala-Lys-Lys-Phe-Val-Lys-S_5-Phe-Lys-NH_2

HPLC: Conditions = solvent A 0.1% TFA/water, solvent B 0.1% TFA/CH_3CN

Gradient = 10-90% gradient of solvent B over 30 min

Purity: 99.6%

HRMS (ESI$^+$) calcd for $C_{108}H_{181}N_{25}O_{17}$ $[M+5H]^{5+}$: 421.0892; found: 421.0873

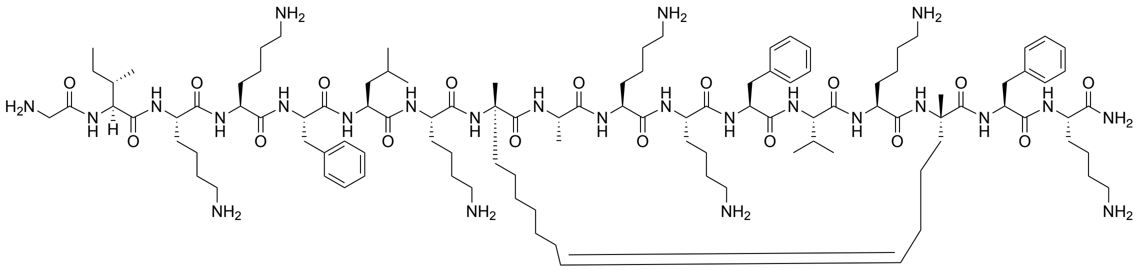

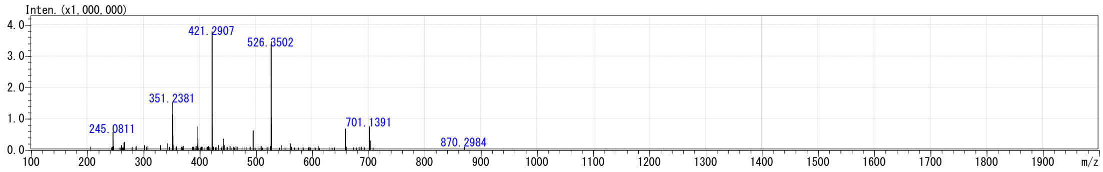

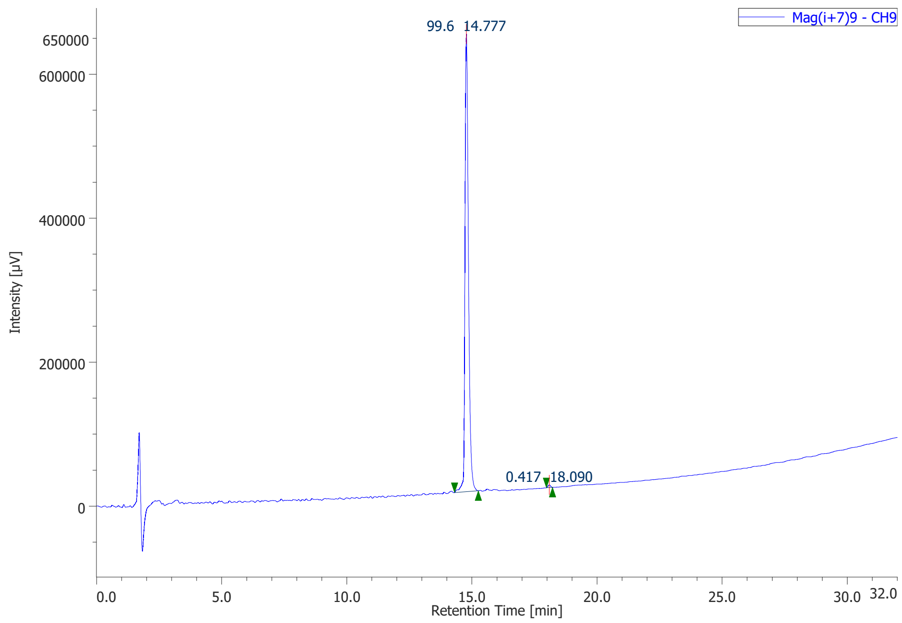

Peptide **9**

H-Gly-Ile-Lys-Lys-Phe-Leu-Lys-Ser-R_8-Lys-Lys-Phe-Val-Lys-Ala-S_5-Lys-NH_2

HPLC: Conditions = solvent A 0.1% TFA/water, solvent B 0.1% TFA/CH_3CN

Gradient = 10-90% gradient of solvent B over 30 min

Purity: 98.8%

HRMS (ESI$^+$) calcd for $C_{102}H_{177}N_{25}O_{18}$ $[M+3H]^{3+}$: 681.1313; found: 681.1311

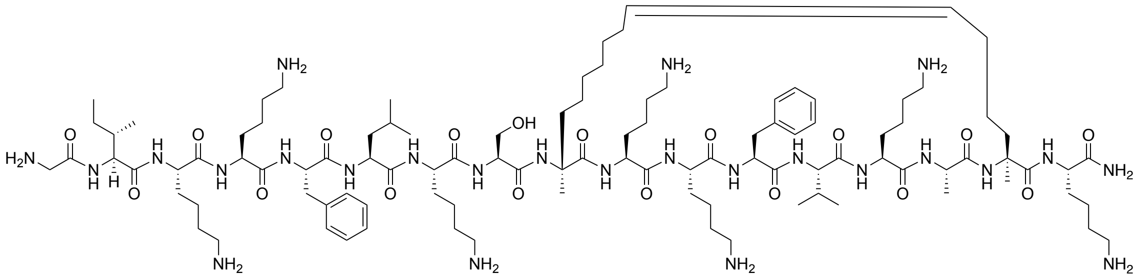

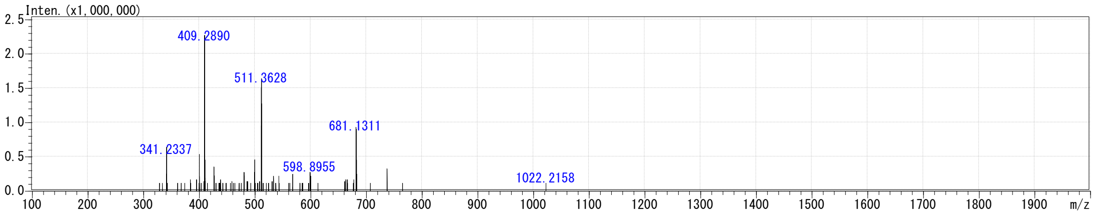

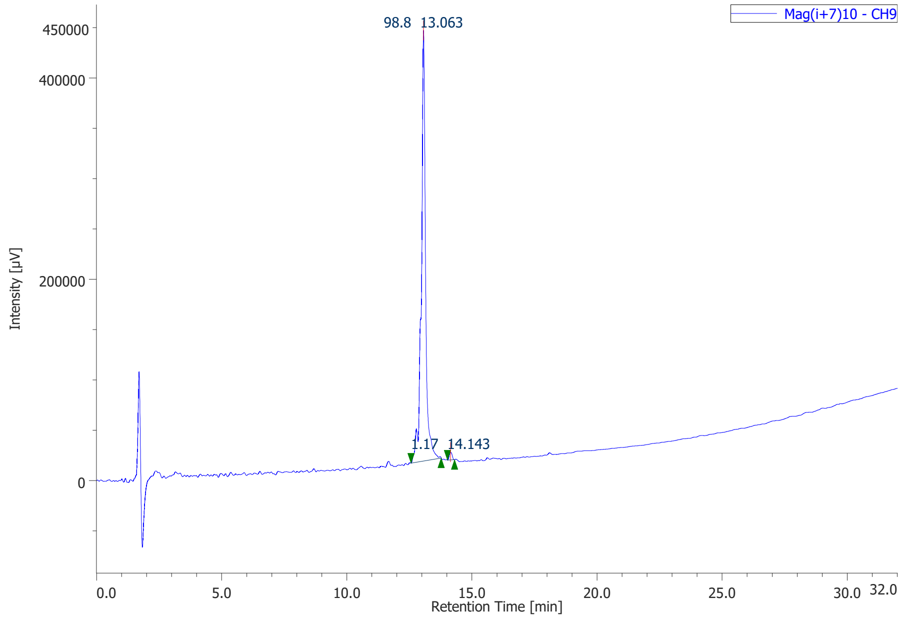

C6-2 (E/Z isomers were detected)

$CH_3(CH_2)_4CO$-S_5-Ile-Lys-Lys-S_5-Leu-Lys-Ser-Ala-Lys-Lys-Phe-Val-Lys-Ala-Phe-Lys-NH_2

HPLC: Conditions = solvent A 0.1% TFA/water, solvent B 0.1% TFA/CH_3CN

Gradient = 10-90% gradient of solvent B over 30 min

Purity: 95.2%

HRMS (ESI^+) calcd for $C_{106}H_{183}N_{25}O_{19}$ $[M+5H]^{5+}$: 423.0902; found: 423.0912

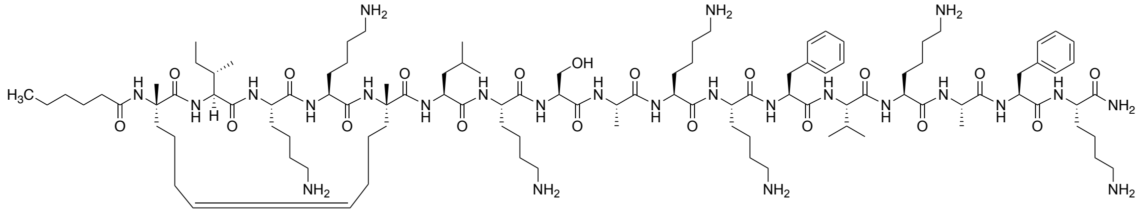

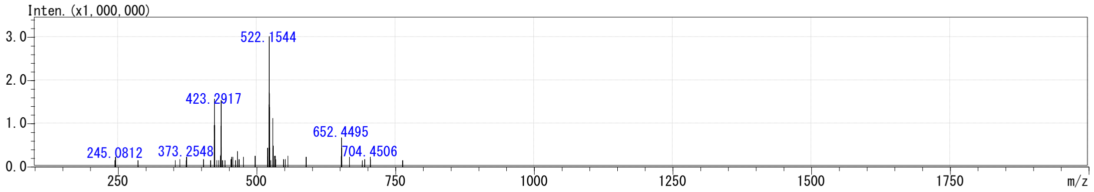

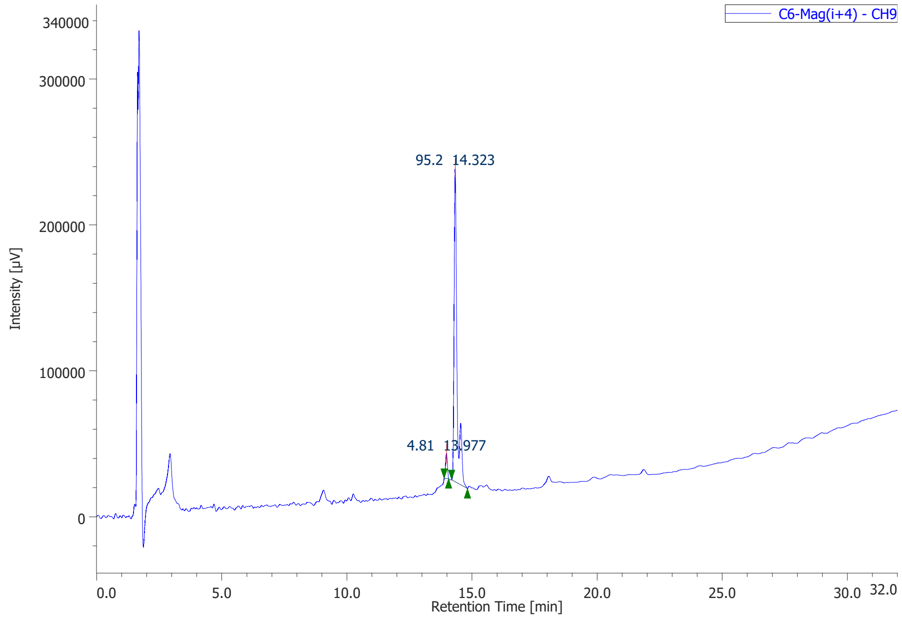

C12-2

$CH_3(CH_2)_{10}CO$-S_5-Ile-Lys-Lys-S_5-Leu-Lys-Ser-Ala-Lys-Lys-Phe-Val-Lys-Ala-Phe-Lys-NH_2

HPLC: Conditions = solvent A 0.1% TFA/water, solvent B 0.1% TFA/CH_3CN

Gradient = 10-90% gradient of solvent B over 30 min

Purity: 98.1%

HRMS (ESI$^+$) calcd for $C_{112}H_{195}N_{25}O_{19}$ $[M+4H]^{4+}$: 549.6343; found: 549.6267

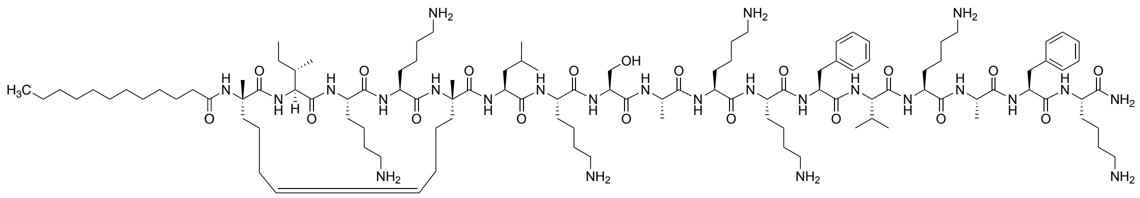

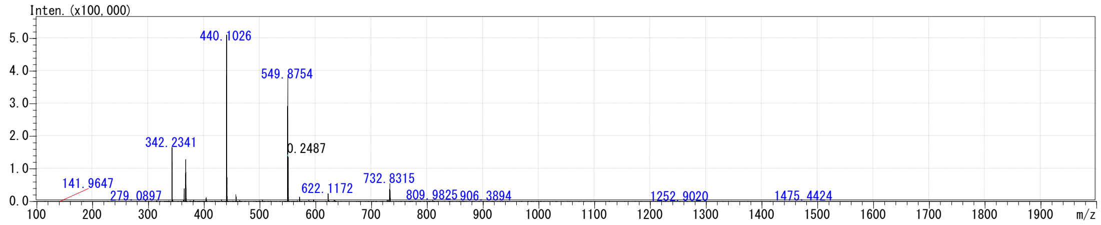

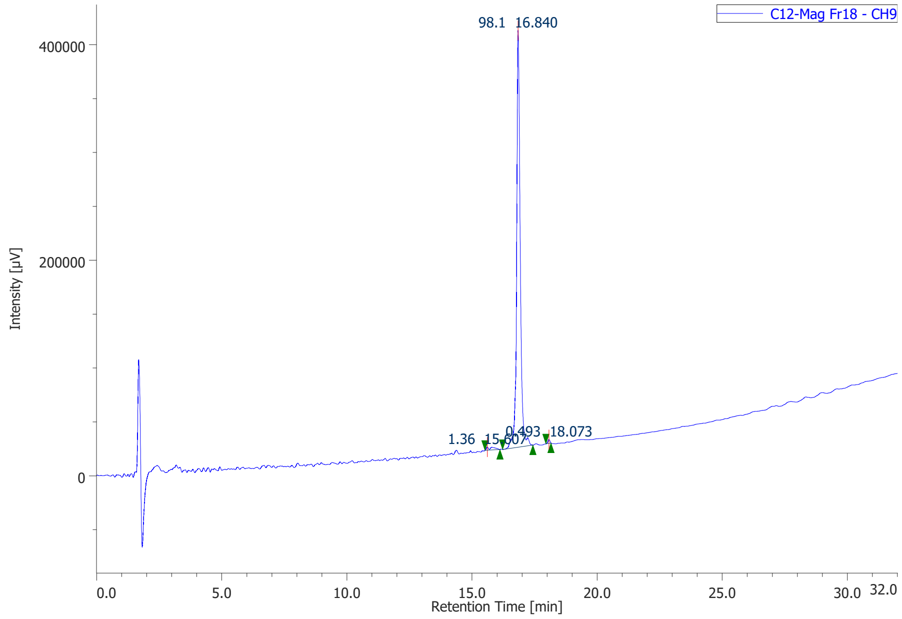

C18-2 (E/Z isomers were detected)

$CH_3(CH_2)_{16}CO$-S_5-Ile-Lys-Lys-S_5-Leu-Lys-Ser-Ala-Lys-Lys-Phe-Val-Lys-Ala-Phe-Lys-NH_2

HPLC: Conditions = solvent A 0.1% TFA/water, solvent B 0.1% TFA/CH_3CN

Gradient = 10-90% gradient of solvent B over 30 min

Purity: 96.2%

HRMS (ESI$^+$) calcd for $C_{118}H_{207}N_{25}O_{19}$ $[M+5H]^{5+}$: 456.7278; found: 456.7152

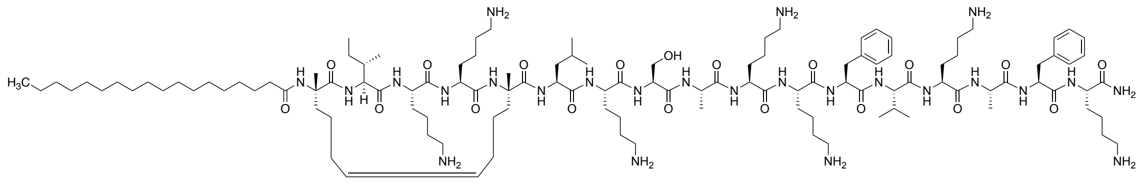

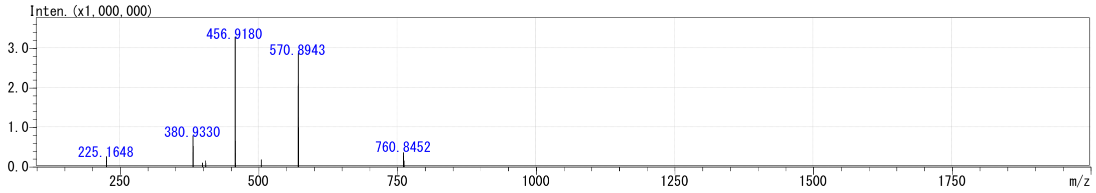

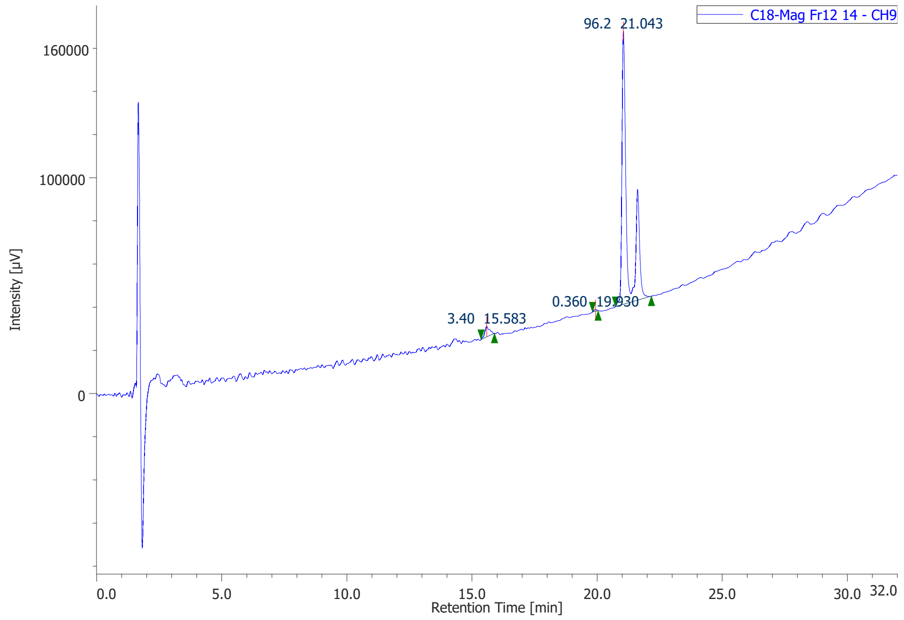

Table S1 The pore forming score for antimicrobial acitivities of peptides 1, 2, and 8 in DOPE/DOPG membrane.

Peptide	1	2	8
Pore stability (%)	48	49	38
Pore diameter (nm)	1.1 ± 0.01	1.2 ± 0.6	0.5 ± 0.01
Charge flux (pC sec^{-1})	32.5 ± 10.3	68.1 ± 13.6	42.9 ± 8.6
Pore forming activity	2.5	1.1	0.4

Table S2 The pore forming score for antimicrobial acitivities of peptides 1, 2, and 8 in DOPC membrane.

Peptide	1	2	8
Pore stability (%)	47	53	60
Pore diameter (nm)	N/A	1.2 ± 0.3	1.7 ± 0.4
Charge flux (pC sec^{-1})	25.3 ± 8.5	21.0 ± 8.4	92.1 ± 16.0
Pore forming activity	0.4	1.1	0.9
